# Predicting 30-Day Readmission for Stroke Using Machine Learning Algorithms: A Prospective Cohort Study

**DOI:** 10.3389/fneur.2022.875491

**Published:** 2022-07-04

**Authors:** Yu-Ching Chen, Jo-Hsuan Chung, Yu-Jo Yeh, Shi-Jer Lou, Hsiu-Fen Lin, Ching-Huang Lin, Hong-Hsi Hsien, Kuo-Wei Hung, Shu-Chuan Jennifer Yeh, Hon-Yi Shi

**Affiliations:** ^1^Department of Healthcare Administration and Medical Informatics, Kaohsiung Medical University, Kaohsiung, Taiwan; ^2^Department of Public Health, College of Medicine, National Cheng-Kung University, Tainan, Taiwan; ^3^Graduate Institute of Technological and Vocational Education, National Pingtung University of Science and Technology, Pingtung, Taiwan; ^4^Department of Neurology, Kaohsiung Medical University Hospital, Kaohsiung, Taiwan; ^5^Department of Neurology, College of Medicine, Kaohsiung Medical University, Kaohsiung, Taiwan; ^6^Division of Neurology, Kaohsiung Veterans General Hospital, Kaohsiung, Taiwan; ^7^Department of Internal Medicine, St. Joseph Hospital, Kaohsiung, Taiwan; ^8^Division of Neurology, Department of Internal Medicine, Yuan's General Hospital, Kaohsiung, Taiwan; ^9^Department of Business Management, National Sun Yat-Sen University, Kaohsiung, Taiwan; ^10^Department of Medical Research, Kaohsiung Medical University Hospital, Kaohsiung, Taiwan; ^11^Department of Medical Research, China Medical University Hospital, China Medical University, Taichung, Taiwan

**Keywords:** 30-day readmission, artificial neural network, feature importance analysis, post-acute care, stroke

## Abstract

**Background:**

Machine learning algorithms for predicting 30-day stroke readmission are rarely discussed. The aims of this study were to identify significant predictors of 30-day readmission after stroke and to compare prediction accuracy and area under the receiver operating characteristic (AUROC) curve in five models: artificial neural network (ANN), K nearest neighbor (KNN), random forest (RF), support vector machine (SVM), naive Bayes classifier (NBC), and Cox regression (COX) models.

**Methods:**

The subjects of this prospective cohort study were 1,476 patients with a history of admission for stroke to one of six hospitals between March, 2014, and September, 2019. A training dataset (*n* = 1,033) was used for model development, and a testing dataset (*n* = 443) was used for internal validation. Another 167 patients with stroke recruited from October, to December, 2019, were enrolled in the dataset for external validation. A feature importance analysis was also performed to identify the significance of the selected input variables.

**Results:**

For predicting 30-day readmission after stroke, the ANN model had significantly (*P* < 0.001) higher performance indices compared to the other models. According to the ANN model results, the best predictor of 30-day readmission was PAC followed by nasogastric tube insertion and stroke type (*P* < 0.05). Using a machine learning ANN model to obtain an accurate estimate of 30-day readmission for stroke and to identify risk factors may improve the precision and efficacy of management for these patients.

**Conclusion:**

Using a machine-learning ANN model to obtain an accurate estimate of 30-day readmission for stroke and to identify risk factors may improve the precision and efficacy of management for these patients. For stroke patients who are candidates for PAC rehabilitation, these predictors have practical applications in educating patients in the expected course of recovery and health outcomes.

## Introduction

Globally, stroke is not only the second leading cause of death, but also the disease with the second largest healthcare burden as estimated in disability-adjusted life-years (1). Previous studies have estimated that as many as 21% of stroke patients are readmitted within 30 days and have found that unplanned Medicare readmission in 2004 estimated in excess of $17 billion in costs (2–4). Furthermore, the mortality rate for 30-day readmission after stroke is more than 2.5 times greater than index admissions and highest among those readmitted for recurrent stroke (2). Additionally, one current study found that ~25.4% of the venous thromboembolism (VTE)-related hospital readmissions occurred within the first 30 days of discharge and they also estimated the mean cost for a hospital readmission with a primary diagnosis of VTE was $18,681; for readmissions with a primary diagnosis of deep vein thrombosis and pulmonary embolism, mean costs were $14,719 and $23,305, respectively (5). Reducing readmission rates among hospitals has become a goal of national healthcare reform.

This prospective study evaluated the use of machine learning algorithms for predicting 30-day readmission after stroke, univariate analysis and feature importance analysis. This study presented a novel opportunity to evaluate the use of post-acute care (PAC) history, demographic characteristics, clinical characteristics, and functional status outcomes as predictors of 30-day readmission in patients with stroke. The results of this study could be used to improve precision and efficacy in managing these patients. These results not only validate the use of similar prediction models for clinical practice in other countries, they also indicate that both PAC and analysis of functional status outcomes should be routinely be integrated in the care for stroke patients.

Although prior works to stratify risk of stroke outcomes have utilized basic statistical models, such as logistic regression been proposed recently, models for predicting readmission have had three major shortcomings. Firstly, recently proposed machine learning models have shown superior area under the receiver operating characteristic (AUROC) curve compared to conventional regression models in predicting 30-day readmission (range: 0.729–0.834 vs. 0.714–0.828, respectively) (6–8). Secondly, proposed forecasting models require use of health insurance claims data, which would not be available in a real-time clinical setting (9). Thirdly, previous studies predicted the risk of readmission do not comprehensively consider baseline patient characteristics, including post-acute care (PAC) history, demographic characteristics, comorbidities, and functional status score (10–12). However, literature on their use for predicting 30-day readmission for stroke is relatively sparse. The current studies regarding to 30-day readmission for patients with cerebrovascular diseases by using machine learning are summarized in Table 1 (6–9, 13–15).

**Table 1:** The studies in predicting 30-day readmission for patients by using machine learning.

**Authors (country)**	**No. of subjects**	**Deep learning algorithms**	**Major findings**
Lineback et al. (USA) (6)	2,855 patients with stroke	1. Logistic regression (LR) 2. Naïve Bayes (NB) 3. Support vector machines (SVM) 4. Random forests (RF) 5. Gradient boosting machines (GBM) 6. Extreme gradient boosting (XGBoost)	Advanced machine learning (ML) methods along with natural language processing (NLP) features out performed logistic regression for all-cause readmission [areas under the curve (AUC), 0.64 vs. 0.58; *P* < 0.001] and stroke readmission prediction (AUC, 0.62 vs. 0.52; *P* < 0.001)
Darabi et al. (USA) (7)	3,184 patients with ischemic stroke	1. Logistic regression (LR) 2. Random forest (RF) 3. Gradient boosting machine (GBM) 4. Extreme gradient boosting (XGBoost) 5. Support vector machines (SVM)	1. GBM provided the highest AUC (0.68), specificity (0.95), and positive predictive value (PPV) (0.33) when compared to the other models 2. In terms of AUC, specificity, and PPV, the LR had poor performance compared to XGBoost and GBM models
Xu et al. (China) (8)	6,070 patients with ischemic stroke	1. Extreme gradient boosting (XGBoost) 2. Logistic regression (LR)	The AUC values of the XGboost model and logistic model for predicting readmission were 0.782 (0.729–0.834) and 0.771 (0.714–0.828), respectively
Sarajlic et al. (Sweden) (9)	149,447 patients with acute myocardial infarction	1. Random forests (RF) 2. k-nearest neighbor (k-NN) 3. Naive Bayes Classifier (NBC) 4. Gradient Boosted Trees (XGBoost) 5. Logistic regression (LR)	The full logistic regression model with 25 predictors had a C-index of 0.67 as compared with the best-performing ML model (Random Forest) with only 10 predictors and a C-index of 0.73
Sharma et al. (Canada) (13)	9,845 patients with heart failure	1. Extreme gradient boosting (XGBoost) 2. Gradient boosting machine (GBM) 3. AdaBoost 4. CatBoost 5. Light gradient boosting machine 6. Support vector machines (SVM) 7. Gaussian naïve Bayes (GNB) 8. Random forest (RF) 9. L1 logistic regression	1. The boosted tree-based ML algorithms had the highest AUC with XGBoost compared to the L1 logistic regression (0.685 vs. 0.591) in predicting 30-day readmission 2. Calibration plots for XGBoost showed that predicted readmission was aligned with observed risks and that low predicted risks were associated with fewer actual outcomes highlighting higher negative predicted values at lower predicted risks
Wang et al. (USA) (14)	47,498 eligible heart failure with reduced ejection fraction patients	1. Logistic regression (LR) 2. Random forest (RF) 3. Extreme gradient boosting (XGBoost)	1. The best AUCs of deep learning (DL) models without a buffer window in predicting heart failure hospitalizations and worsening heart failure events in the total patient cohort were 0.977 and 0.972, respectively 2. The best AUCs in predicting 30-day readmission in all adult patients were 0.597 and 0.614, respectively 3. For all outcomes assessed, the DL approach outperformed traditional machine learning (ML) models
Amritphale et al. (USA) (15)	16,745 patients with carotid artery stenting	1. Logistic regression (LR) 2. Support vector machine (SVM) 3. Deep neural network (DNN) 4. Random forest (RF) 5. Decision tree (DT)	1. The artificial intelligence machine learning DNN prediction model has a C-statistic value of 0.79 in predicting the patients who might have all-cause unplanned readmission within 30 days of the index carotid artery stenting discharge 2. The DNN model showed a significant higher receiver operating characteristic (ROC; 0.802 vs. 0.680, 0.670, 0.607, and 0.586, respectively) and precision-recall (0.383 vs. 0.140, 0.140, 0.380, and 0.269, respectively) than the LR, SVM, RF, and DT in predicting 30-day readmission among patients with carotid artery stenting

To reduce 30-day readmission after stroke and subsequent mortality, identifying factors that predict readmission is crucial. Determining the risk factors for 30-day readmission may be useful for developing policies for preventing readmission after stroke. Therefore, the aims of this study were to compare forecasting accuracy in the artificial neural network (ANN), K nearest neighbor (KNN), random forest (RF), support vector machine (SVM), naive Bayes classifier (NBC) and Cox regression (COX) models and to explore significant predictors of readmission within 30 days after stroke. The key contributions of this study can be summarized as follows:

- Advances in artificial intelligence have been applied in clinical practice. However, machine learning algorithms have not been used to predict 30-day readmission for patients with stroke mainly because of the high complexity of prediction algorithms relative to diagnostic algorithms.
- The proposed machine learning algorithms exhibit strong potential for use in predicting readmission within 30 days after stroke.
- A feature importance analysis was also performed to determine the significance of the selected input variables.

## Materials and Methods

### Study Design and Patients

**Figure 1:**
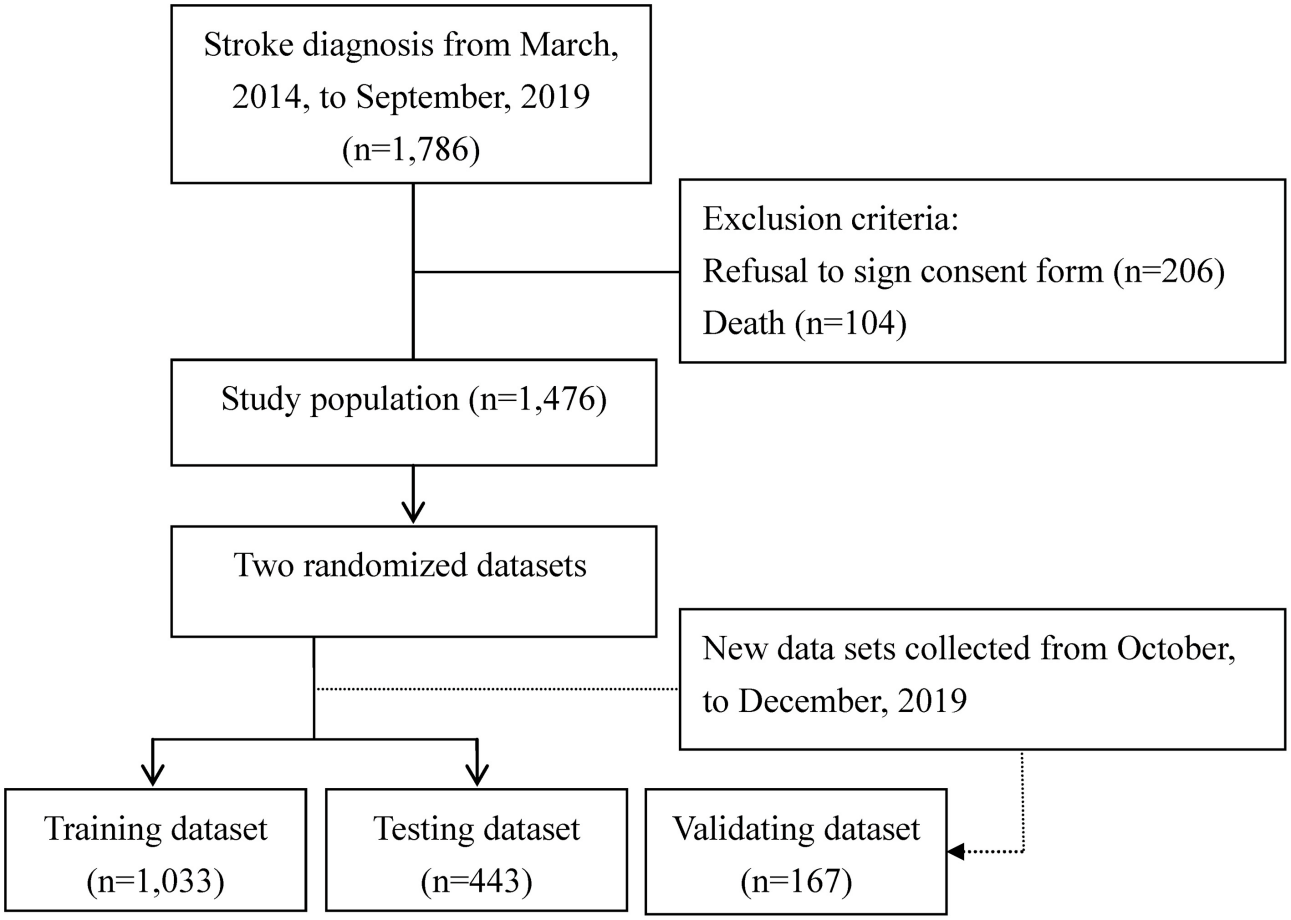
Flowchart of the study.

**Figure 2:**
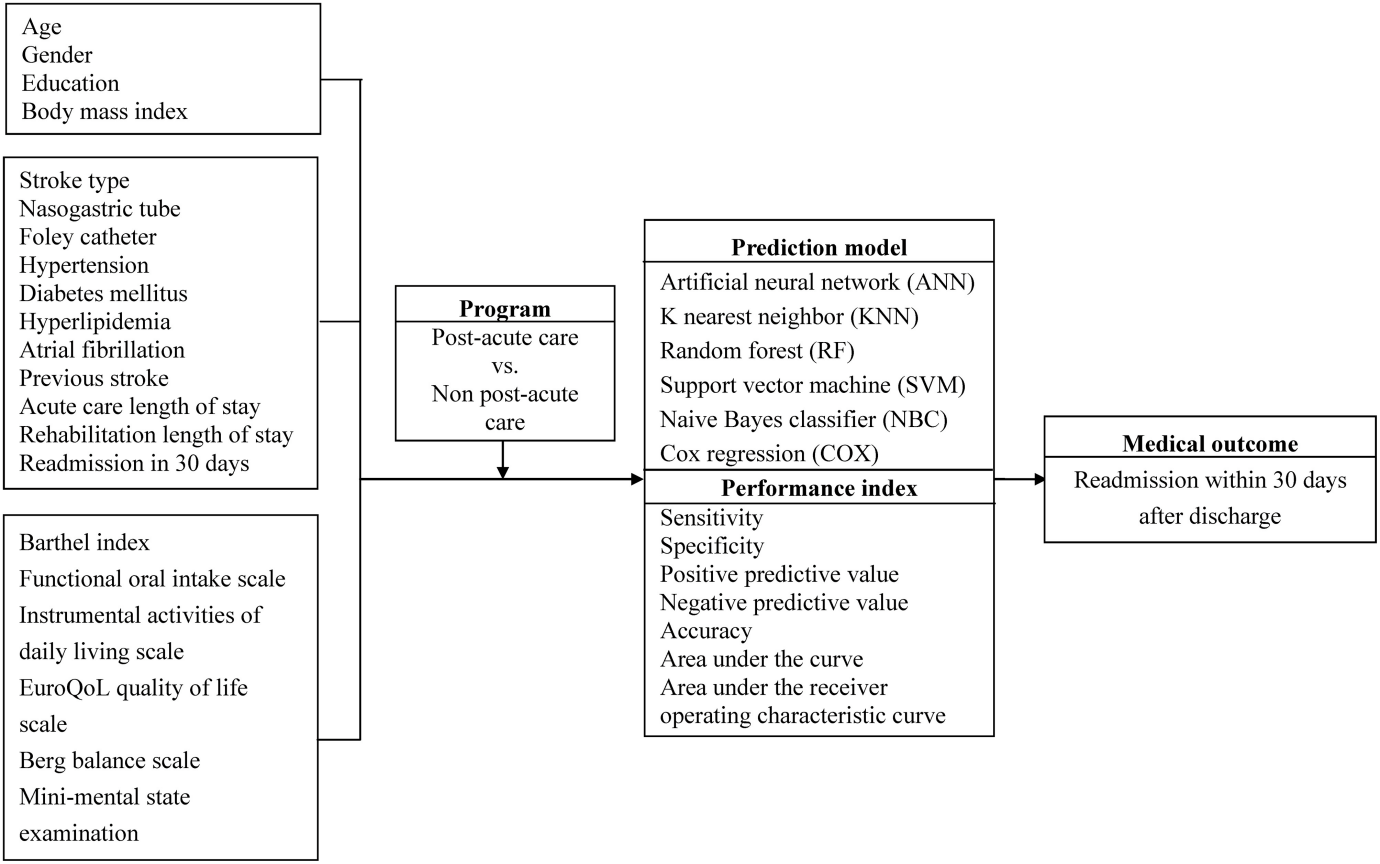
Conceptual framework of the proposed method for predicting readmission within 30 days after stroke.

The subjects of this prospective cohort study were 1,476 patients with a record of an ICD-9-CM (433.01, 433.10, 433.11, 433.21, 433.31, 433.81, 433.91, 434.00, 434.01, 434.11, 434.91 and 436 for ischemic stroke; 430 and 431 for hemorrhagic stroke), ICD-10 (I60–I62 were used to identify hemorrhagic stroke; I63 was used for ischemic stroke), and a history of admission to the PAC ward at one of four hospitals (three regional hospitals and one district hospital) or to a traditional non-PAC ward at one of two medical centers in south Taiwan between March, 2014, and September, 2019. The enrollment criteria were patients hospitalized for their first-ever stroke who were examined within 30 days with computed tomography (CT) or magnetic resonance imaging (MRI) and a Modified Rankin Scale (MRS) score of 2 to 4. Scores for the MRS range from 0 to 6, and a high MRS score indicates a high severity of disability. Patients were excluded if PAC beds were unavailable at the participating hospitals or if they had been transferred to PAC wards at other hospitals. In this scale, absence of symptoms is scored as 0. No significant disability, slight disability moderate disability moderately severe disability, and severe disability is scored as 1, 2, 3, 4 and 5, respectively (16). Another 167 stroke patients were recruited from October to December, 2019 (Figure 1). Figure 2 also depicts the conceptual framework of the proposed method for predicting readmission within 30 days after stroke. The study protocol was approved by the institutional review board at Kaohsiung Medical University Hospital (KMUH-IRB-20140308), and written informed consent was obtained from each participant.

### Instruments and Potential Predictors

Functional disability was measured using the 10-item Barthel Index (BI) (17). The BI measures functional disability in terms of inability to perform certain daily life activities (e.g., dressing, performing self-care, and walking up and down stairs). A BI score of 10 indicates complete independence. In stroke patients who had dysphagia, functional oral intake was assessed with the Functional Oral Intake Scale (FOIS) (18), in which swallowing function is classified on a scale from 1 (nil by mouth) to 7 (total oral diet without restriction). Cognitive status was quantitatively assessed with the Mini-mental State Examination (MMSE) (19). The MMSE includes tests for orientation, memory, attention, calculation, language, and construction functions where higher scores indicate better functional status (total score range, 0–30). The Instrumental Activities of Daily Living (IADL) scale is most useful for assessing current function and improvement or deterioration in function over time (20). When the IADL scale is administered in women, all eight domains for function are scored. In men, the domains of food preparation, housekeeping, and laundering are not scored. The EuroQoL Quality of Life Scale (EQ-5D-3L) measures the total health state of the subject based on a self-assessment of 5 items: mobility, self-care, usual activities, pain or discomfort, and anxiety or depression (21). Each EQ-5D-3L item is scored as 1 (no problem), 2 (some problem), or 3 (extreme problem). The 14-item Berg Balance Scale (BBS) is used to measure functional balance (22). Each item is rated from 0 (poor) to 4 (good), and the maximum score is 56. The Chinese versions of all instruments used in this study have been validated and used extensively in both clinical practice and research (17, 23).

A research assistant collected the following data from medical records after index discharge: PAC program (PAC group or non-PAC group), patient attributes (age, gender, education, and BMI), clinical attributes [stroke type, NG tube, Foley catheter, hypertension, diabetes mellitus (DM), hyperlipidemia, atrial fibrillation, previous stroke, acute care LOS, and rehabilitation ward LOS]. In multivariate analysis, the potential predictors were the independent variables, and 30-day readmission was the dependent variable.

### Machine Learning Algorithms

Machine learning algorithms are effective tools for identifying and classifying readmission within 30 days after discharge in patients with stroke. Previous studies have successfully used machine learning to classify stroke according to characteristics such as cardiac source and gait in various scenarios (24, 25). In the present study, machine learning algorithms used to predict 30-day readmission in patients with stroke included ANN, KNN, RF, SVM, NBC and COX models.

### Statistical Analysis

The unit of analysis in this study was the individual patient with stroke. Statistical analysis was performed in the following steps. In the first step, the statistical significance of continuous variables was tested by one-way analysis of variance, and that of categorical variables was tested by Fisher exact analysis. Univariate analyses were performed to identify significant predictors (*P* < 0.05). In the second step, data for the study cohort of 1,476 subjects were randomly divided into two datasets: a training dataset containing data for 1,033 subjects (70%), which was used for model development, and a testing dataset containing data for 443 subjects (30%), which was used for internal validation. A validation dataset containing data for another 167 patients enrolled after September, 2019, was used for external validation. To identify the optimal hyper-parameters for the machine learning algorithms, we applied Bayesian optimization using the expected improvement as the acquisition function (26). To perform the hyperband method of optimization and to test different combinations of hyper-parameters, we used Optuna version 2.10.0 (27). A total of 1,000 trials were conducted, and the parameters with the greatest area under the receiver operating characteristic curve were saved. Additionally, since data used for model fitting tended to overestimate model performance on unseen subjects, we coupled 10-fold cross-validation (28) with the logistic loss metric to measure the generalizability of the model to unseen subjects during model selection. A total of six machine-learning classifiers were constructed in the training dataset and tested in the validating dataset. A confusion matrix is used to describe and visualize the performance of the machine learning algorithm classifier and also to provide insight on what the model misclassifies. In the present study, the performance of the machine learning algorithms for the best classification task was evaluated in terms of confusion matrix-based performance measuring metrics including sensitivity, specificity, positive predictive value (PPV), negative predictive value (NPV), and accuracy. In order to evaluate and select the most accurate machine learning algorithms, we used a confusion matrix and calculated the percentage of sensitivity, specificity, and accuracy of each forecasting model. In addition, the performance of the machine learning algorithms in the present study was also evaluated by the receiver operating characteristic (ROC) curve and the area under the ROC curve (AUROC). The independent variables fitted to the forecasting models were significant predictors of 30-day readmission, and the dependent variable was 30-day readmission. After model training, model outputs were collected for each testing dataset. In the third step, bootstrapping, a machine learning technique, which involves taking random samples from the dataset with re-selection of 1,000 resamples was used to compare different machine learning algorithms employing the performance indices and the 95% confidence intervals. We used paired *t*-test to identify performance indices that significantly differed between the two models.

In the fourth and final step, feature selection method was calculated by using an algorithm to obtain an importance score for each potential predictor in the dataset (29). Feature importance analysis provides information about how each feature contributes to model prediction accuracy. The final weight of each feature is calculated by averaging the decrease in model accuracy after random permutation of the feature values within a testing set. Permutation of an important feature should decrease the score whereas permutation of a feature that is not very important to model prediction accuracy should increase the score. To obtain robust results with our small dataset, the train-test split was performed with a repeated stratified K fold cross validation. This technique has two advantages: first, it is model-agnostic; second, it can be performed repeatedly with different feature permutations. All statistical analyses were performed using the STATISTICA 13.0 software package (StatSoft, Inc., Tulsa, OK, USA). All statistical tests were two-sided; a *P*-value < 0.05 was considered statistically significant.

## Results

### Study Characteristics

Table 1 shows that 1,283 patients (86.9%) joined the per-diem PAC program and the remaining patients selected the fee-for-service non-PAC program. The patients with stroke had a mean age of 65.5 years (standard deviation, SD 13.0 years), and most (62.5%) patients were male. During the study period, 120 patients with stroke were readmitted within 30 days. In univariate analysis, PAC program, age, gender, education, body mass index (BMI), stroke type, nasogastric (NG) tube, Foley, hypertension, diabetes mellitus (DM), hyperlipidemia, atrial fibrillation, previous stroke, acute care length of stay (LOS), rehabilitation LOS and functional status score before rehabilitation were significantly associated with 30-day readmission (*P* < 0.05). These significant predictors were included in the forecasting models (Table 2).

**Table 2:** Baseline characteristics of the study population (*N* = 1,476).

**Variables**	Mean ±SD or *N* (%)^*^
**Post-acute care**	
No	193 (13.1)
Yes	1,283 (86.9)
**Patient attributes**	
Age (years)	65.5 ± 13.0
Gender	
Female	554 (37.5)
Male	922 (62.5)
Education (years)	8.9 ± 2.1
Body mass index (kg/m^2^)	24.0 ± 2.6
**Clinical attributes**	
Stroke type
Ischemic	1,224 (82.9)
Hemorrhagic	252 (17.1)
Nasogastric tube	
No	1,187 (80.4)
Yes	289 (19.6)
Foley catheter	
No	1,342 (90.9)
Yes	134 (9.1)
Hypertension	
No	449 (30.4)
Yes	1,027 (69.6)
Diabetes mellitus	
No	906 (61.4)
Yes	570 (38.6)
Hyperlipidemia	
No	967 (65.5)
Yes	509 (34.5)
Atrial fibrillation	
No	1,354 (91.7)
Yes	122 (8.3)
Previous stroke	
No	1,250 (84.7)
Yes	226 (15.3)
Acute care length of stay (days)	15.2 ± 9.0
Rehabilitation length of stay (days)	44.9 ± 21.2
Readmission in 30 days
No	1,356 (91.9)
Yes	120 (8.1)
**Functional status scores before rehabilitation**	
BI score	39.0 ± 23.7
FOIS score	5.5 ± 2.1
EQ5D score	10.4 ± 1.9
IADL score	1.2 ± 1.1
BBS score	15.6 ± 15.8
MMSE score	19.4 ± 8.9

* *Data are frequencies (percentages), as indicated, for categorical variables and mean ± standard deviation for continuous variables of baseline characteristics*.

*SD, standard deviation; BI, Barthel Index; FOIS, Functional Oral Intake Scale; EQ-5D, EuroQoL Quality of Life Scale; IADL, Instrumental activities of Daily Living Scale; BBS, Berg Balance Scale; MMSE, Mini-Mental State Examination*.

### Comparison of Forecasting Models

**Figure 3:**
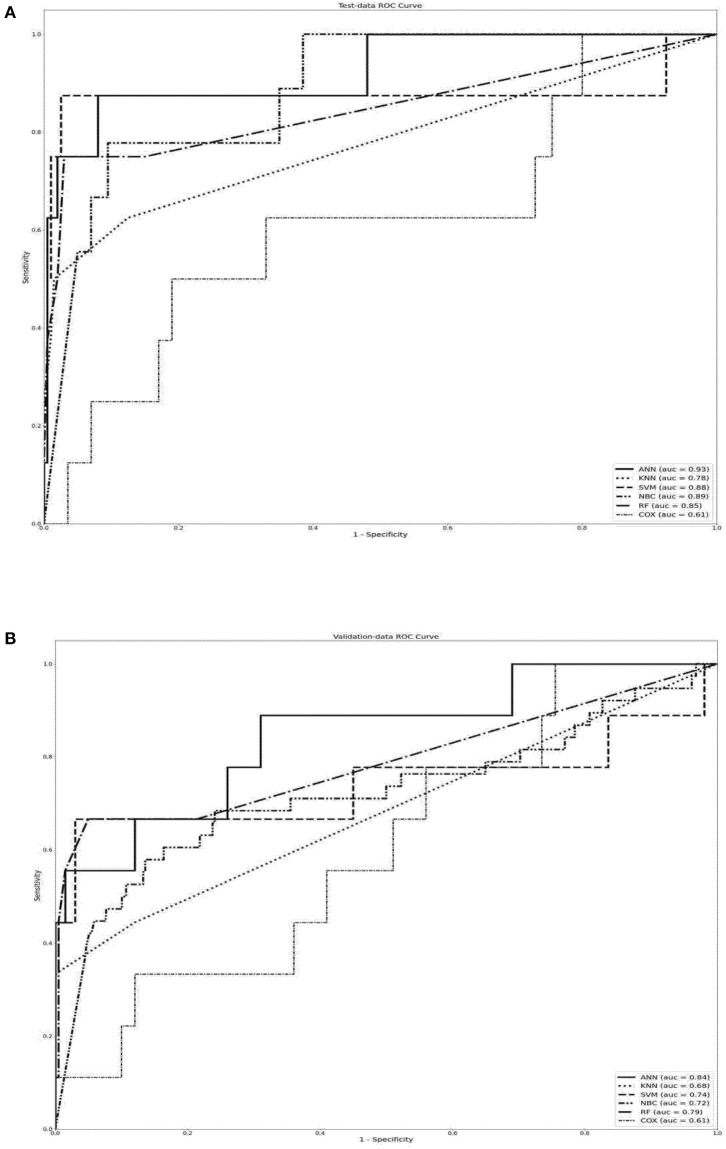
Performance indices of forecasting models used to predict 30-day readmission in patients with stroke when using **(A)** training dataset, **(B)** testing dataset. The box plot shows the median (centers) and interquartile range (borders). In analyses of accuracy and AUROC, the ANN model had significantly higher values compared to other forecasting models (*P* < 0.001). AUROC, area under the receiver operating characteristics; ANN, artificial neural network.

Significant predictors of 30-day readmission did not significantly differ between the training and testing datasets; therefore, samples were compared between the training and testing datasets to increase reliability of the validation results (Table 3). We used grid search to find the best hyperparameters for the neural networks. We searched for the following hyperparameters: the number of hidden layers (in the range of 1–6), the number of hidden neurons in each layer (in the range of 1–512), activation functions (“relu,” “logistic sigmoid”), and learning rate (in the range of 0.01–0.001). We used adam optimizer, constant learning rate, and the regularization rate of alpha = 0.01. The SVM model was configured with linear kernel, and regularization parameter *C* = 1.0. The RF model is an ensemble learning method combined of multiple decision tree predictors that are trained based on random data samples and feature subsets. We configured the RF algorithm with two trees in the forest. Hyperparameter optimization was then performed to improve the performance of the compact model, and the machine learning algorithms with the greatest AUROC values in 1,000 trials were obtained. Table 4 lists the final hyperparameter settings. The data in Table 5 indicate that the ANN model compared to KNN, RF, SVM, NBC, and COX models had significantly (*P* < 0.001) higher sensitivity, specificity, PPV, NPV, accuracy, and AUC values. Similar results also were shown in dataset for testing simultaneously. The receiver operating characteristic (ROC) curve results in Figure 3 show that the ANN model had significantly higher ROC values compared to other forecasting models (*P* < 0.001).

**Table 3:** Univariate analysis of selected risk factors for 30-day readmission in patients with stroke (*N* = 1,476).

**Variables**	**Statistics**	*P*-value^*^
**Post-acute care (yes vs. no)**	52.074	<0.001
**Patient attributes**		
Age (years)	7.890	0.005
Gender (female vs. male)	23.657	<0.001
Education (years)	10.870	<0.001
Body mass index (kg/m^2^)	7.944	0.005
**Clinical attributes**		
Stroke type (ischemic vs. hemorrhagic)	32.053	<0.001
Nasogastric tube (yes vs. no)	49.361	<0.001
Foley catheter (yes vs. no)	5.590	0.018
Hypertension (yes vs. no)	4.564	0.033
Diabetes mellitus (yes vs. no)	7.324	0.007
Hyperlipidemia (yes vs. no)	5.777	0.016
Atrial fibrillation (yes vs. no)	6.114	0.013
Previous stroke (yes vs. no)	6.899	0.009
Acute care length of stay, days	30.008	<0.001
Rehabilitation length of stay, days	26.508	<0.001
**Functional status score before rehabilitation**		
BI score	37.494	<0.001
FOIS score	26.508	<0.001
EQ5D score	16.712	<0.001
IADL score	22.726	<0.001
BBS score	14.903	<0.001
MMSE score	34.665	<0.001

* *One-way analysis of variance and Fisher exact analysis were performed to assess for associations between the variables and 30-day readmission*.

*BI, Barthel Index; FOIS, Functional Oral Intake Scale; EQ-5D, EuroQoL Quality of Life Scale; IADL, Instrumental Activities of Daily Living Scale; BBS, Berg Balance Scale; MMSE, Mini-Mental State Examination*.

**Table 4:** Hyper-parameters and final settings in all machine learning algorithms.

**Algorithms**	**Hyper-parameters**	**Settings**
Artificial neural network (ANN)	Hidden layers	6
	Hidden neuron	512-256-128-64-32-1
	Learning rate^*^	0.001
K nearest neighbor (KNN)	Neighbors	5
Support vector machine (SVM)	C_penalty_	1.0
	Gamma	1/[*n*_features * X.var()]
Naive Bayes classifier (NBC)	Alpha	1.0
Random forest (RF)	Estimators	100
	Split_min_	2
	leaf_min_	1
Cox regression (COX)	–	–

* *Optimizer algorithm using Adam*.

**Table 5:** Comparison of 1,000 pairs of forecasting models for predicting 30-day readmission in patients with stroke (*N* = 1,476).

**Model**	**Sensitivity**	**Specificity**	**PPV**	**NPV**	**Accuracy**	**AUC**
**Training dataset (*****n*** **=** **1,033)**						
ANN (95% CI)	0.73 (0.65, 0.82)	0.98 (0.96, 0.99)	0.88 (0.84, 0.92)	0.77 (0.70, 0.84)	0.92 (0.89, 0.95)	0.94 (0.91,0.97)
KNN (95% CI)	0.59 (0.50, 0.68)	0.86 (0.82, 0.90)	0.56 (0.47, 0.65)	0.64 (0.56, 0.72)	0.83 (0.78, 0.88)	0.76 (0.68, 0.84)
RF (95% CI)	0.70 (0.64, 0.76)	0.92 (0.87, 0.97)	0.79 (0.75, 0.83)	0.71 (0.64, 0.78)	0.88 (0.84, 0.92)	0.85 (0.80, 0.90)
SVM (95% CI)	0.49 (0.39, 0.59)	0.96 (0.93, 0.99)	0.76 (0.68, 0.84)	0.62 (0.54, 0.70)	0.89 (0.85, 0.93)	0.74 (0.66, 0.82)
NBC (95% CI)	0.48 (0.38, 0.59)	0.96 (0.93, 0.99)	0.50 (0.40, 0.60)	0.69 (0.61, 0.77)	0.81 (0.75, 0.87)	0.73 (0.65, 0.81)
COX (95% CI)	0.51 (0.42, 0.61)	0.97 (0.95, 0.99)	0.77 (0.69, 0.85)	0.71 (0.63, 0.79)	0.85 (0.80, 0.90)	0.88 (0.83, 0.93)
*P*-value^*^	<0.001	<0.001	<0.001	<0.001	<0.001	<0.001
**Testing dataset (*****n*** **=** **443)**						
ANN (95% CI)	0.70 (0.62, 0.78)	0.97 (0.95, 0.99)	0.89 (0.85, 0.93)	0.82 (0.76, 0.88)	0.93 (0.90, 0.96)	0.89 (0.85, 0.93)
KNN (95% CI)	0.53 (0.44, 0.62)	0.88 (0.84, 0.92)	0.60 (0.51, 0.69)	0.71 (0.63, 0.79)	0.71 (0.63, 0.79)	0.81 (0.75, 0.87)
RF (95% CI)	0.69 (0.62, 0.76)	0.94 (0.92, 0.96)	0.85 (0.82, 0.88)	0.79 (0.76, 0.82)	0.88 (0.84, 0.92)	0.87 (0.83, 0.91)
SVM (95% CI)	0.53 (0.44, 0.62)	0.93 (0.90, 0.96)	0.75 (0.67, 0.82)	0.78 (0.71, 0.85)	0.82 (0.73, 0.89)	0.80 (0.74, 0.86)
NBC (95% CI)	0.50 (0.40, 0.60)	0.93 (0.90, 0.96)	0.63 (0.54, 0.72)	0.79 (0.72, 0.86)	0.83 (0.76, 0.90)	0.84 (0.78, 0.90)
COX (95% CI)	0.54 (0.45, 0.64)	0.96 (0.94, 0.98)	0.88 (0.83, 0.93)	0.61 (0.53, 0.69)	0.87 (0.82, 0.92)	0.87 (0.82, 0.92)
*P*-value^*^	<0.001	<0.001	<0.001	<0.001	<0.001	<0.001

*ANN, artificial neural network; KNN, K nearest neighbor; RF, random forest; SVM, support vector machine; NBC, naive Bayes classifier; COX, Cox regression; PPV, positive predictive value; NPV, negative predictive value; AUC, area under the curve; CI, confidence interval*.

* *The P-value is the statistical significance of the forecasting models and performance indices calculated using a Chi-squared test*.

### Significant Predictors in the ANN Model

**Figure 4:**
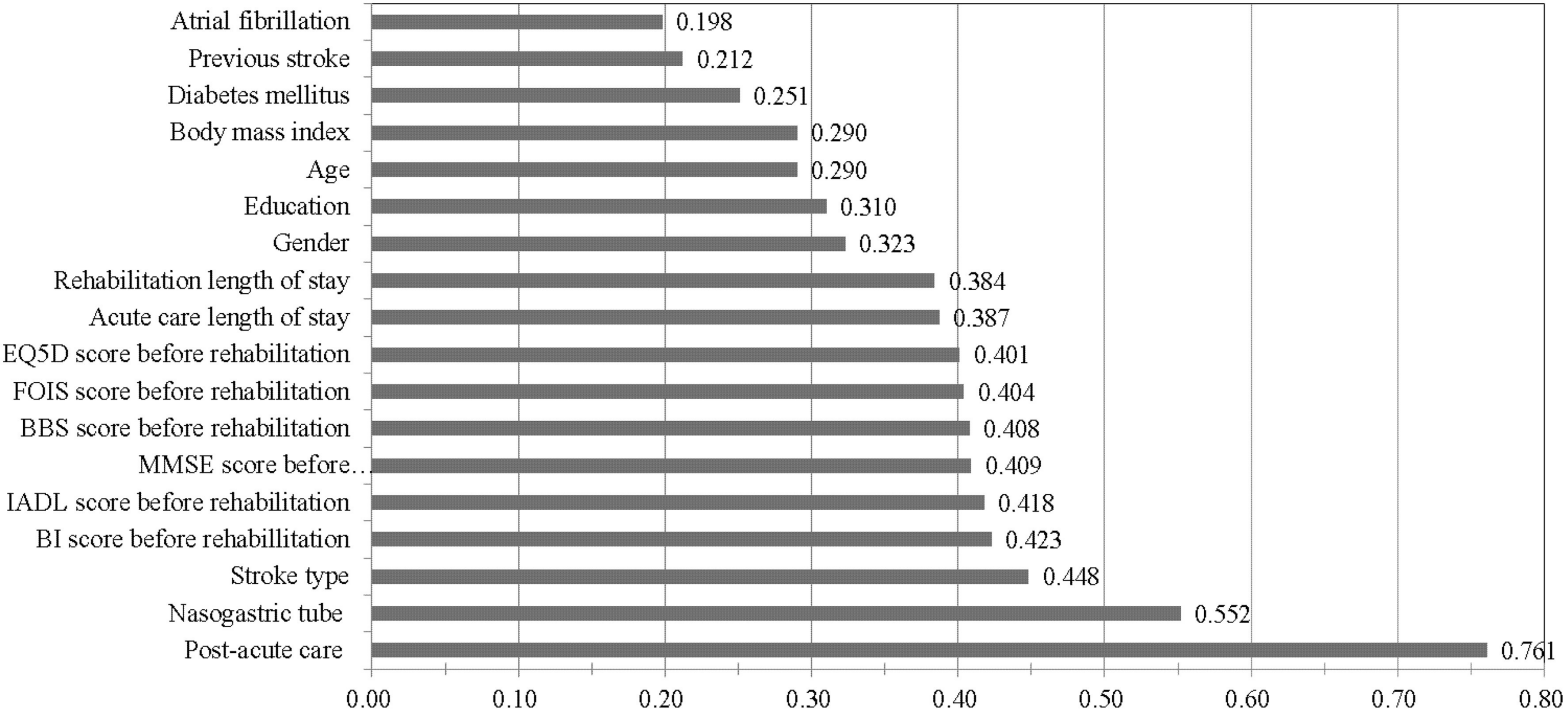
A permutation importance analysis of artificial neural network model in predicting 30-day readmission in patients with stroke. BI, Barthel Index; IADL, Instrumental Activities of Daily Living; MMSE, Mini-Mental State Examination; BBS, Berg Balance Scale; FOIS, Functional Oral Intake Scale; EQ-5D, EuroQoL Quality of Life Scale.

Figure 4 shows the feature importance analysis results for the ANN model. The VSR value for predicting 30-day readmission in stroke patients was highest for PAC (permutation importance = 0.761) followed by NG tube (0.552), stroke type (0.448), BI score before rehabilitation (0.423), IADL score before rehabilitation (0.418), MMSE score before rehabilitation (0.409), BBS score before rehabilitation (0.408), FOIS score before rehabilitation (0.404), EQ5D score before rehabilitation (0.401), and others.

### Sensitivity Analysis

Next, the validating dataset of 167 subjects was used to compare the predictive accuracy of the models. Table 6 also compares the performance indices obtained in external validation of the ANN, KNN, RF, SVM, NBC and COX models. For predicting 30-day readmission, the ANN model consistently achieved significantly higher performance indices (*P* < 0.001).

**Table 6:** Comparative performance indices of forecasting models when using 167 new validating datasets to predict 30-day readmission in patients with stroke.

**Models**	**Sensitivity**	**Specificity**	**PPV**	**NPV**	**Accuracy**	**AUC**
ANN (95% CI)	0.74 (0.66, 0.82)	0.97 (0.95, 0.99)	0.89 (0.85, 0.94)	0.87 (0.82, 0.92)	0.93 (0.90, 0.96)	0.94 (0.91, 0.97)
KNN (95% CI)	0.50 (0.40, 0.49)	0.87 (0.83, 0.91)	0.61 (0.52, 0.70)	0.70 (0.62, 0.78)	0.80 (0.74, 0.86)	0.83 (0.78, 0.88)
RF (95% CI)	0.70 (0.66, 0.74)	0.95 (0.91, 0.98)	0.84 (0.80, 0.88)	0.85 (0.81, 0.89)	0.90 (0.87, 0.93)	0.90 (0.86, 0.94)
SVM (95% CI)	0.51 (0.41, 0.61)	0.96 (0.94, 0.98)	0.76 (0.69, 0.83)	0.79 (0.72, 0.87)	0.88 (0.84, 0.92)	0.81 (0.76, 0.86)
NBC (95% CI)	0.50 (0.40, 0.60)	0.93 (0.90, 0.96)	0.61 (0.52, 0.70)	0.80 (0.73, 0.87)	0.84 (0.79, 0.89)	0.80 (0.75, 0.85)
COX (95% CI)	0.58 (0.49, 0.67)	0.92 (0.89, 0.95)	0.84 (0.78, 0.90)	0.69 (0.61, 0.77)	0.88 (0.84, 0.92)	0.88 (0.84, 0.92)
*P-*value^*^	<0.001	<0.001	<0.001	<0.001	<0.001	<0.001

*ANN, artificial neural network; KNN, K nearest neighbor; RF, random forest; SVM, support vector machine; NBC, naive Bayes classifier; COX, Cox regression; PPV, positive predictive value; NPV, negative predictive value; AUC, area under the curve; CI, confidence interval*.

* *The P-value is the statistical significance of the forecasting models and the performance indices calculated using a Chi-squared test*.

## Discussion

Accuracy in predicting 30-day readmission in patients with stroke was compared among five forecasting models. For a given set of clinical inputs, the ANN model clearly had superior forecasting accuracy compared to the other four. Notably, our prospective study collected longitudinal data from six different medical institutions, which provided a real-world depiction of current treatment for patients with stroke. In contrast, previous works have used data from a single medical center (10–13). Moreover, using registry data obtained from six hospitals mitigated the potential for referral bias or bias caused by analyzing the practices of a single physician or a single institution (30, 31).

Recent works have demonstrated the superior performance of machine learning-based models for predicting stroke outcomes (24, 25). One advantage of using an ANN model is that it enables appropriate and accurate processing of inputs that are incomplete or inputs that introduce noise (9, 32). Another advantage of ANN models, whether linear or non-linear, is their good performance in/effectiveness for analyzing large-scale medical databases constructed using data that are highly correlated but not normally distributed. The high robustness of the ANN model has been demonstrated in many clinical applications, particularly predicting prognosis in various diseases (32). In performance comparisons of the five models in this study, expanding the number of potential predictors apparently improved the performance of the ANN model in systematic analysis of outcome in various diseases.

Our current results indicate that ANN models can use clinical outcome data for predicting 30-day readmission after stroke. Prospective prediction performance and cross-validation performance were adequate when subjects were familiar with the task and when information from the previous test session was made available. However, larger scale studies are still needed to validate this approach.

A permutation importance analyses of the weights of significant predictors of 30-day readmission for stroke revealed that the best predictor was PAC. This finding is consistent with earlier reports that, in comparisons of independent predictors, PAC is the best predictor of stroke outcome, including overall treatment cost, functional status after stroke, and duration of hospital stay before transfer to rehabilitative ward (30, 33). In a quasi-experimental study of stroke patients, Wang et al. (30) investigated the longitudinal impact of PAC on functional status. The authors concluded that multidisciplinary rehabilitative PAC delivered on a per-diem basis substantially improved functional status compared to standard rehabilitation. Another study performed in a nationwide stroke cohort compared mortality and numerous functional domains between a PAC group and a well-matched non-PAC group (34). The PAC group had significantly lower 90-day hospital readmissions and stroke-related readmissions compared to the non-PAC group.

Dennis et al. (35) reported that, compared to nasogastric feeding, percutaneous endoscopic gastrostomy was associated with higher risk of death or poorer outcomes at 6 months after stroke. However, Ho et al. (36) noted that prolonged (i.e., longer than 2 weeks) nasogastric tube feeding was significantly associated with pneumonia and mortality. In the current study, NG tube insertion before rehabilitation was significantly associated with 30-day readmission (*P* < 0.001). During the study period, no patient with stroke required NG tube insertion after rehabilitation.

Compared to other stroke types, hemorrhagic stroke is reportedly associated with higher severity and with higher overall mortality in the first 3 months after stroke (37, 38). The current study further revealed that hemorrhagic stroke has a higher 30-day readmission rate for ischemic stroke.

This prospective observational cohort study of patients with stroke in Taiwan analyzed data from patients treated at six healthcare institutions. The predictive accuracy of the ANN model developed in this study outperformed the other four models in identifying predictors of 30-day readmission. Three implications of this study are noted. First, the proposed ANN model may be useful for guiding the clinical care of patients with stroke. Second, healthcare administrators and managers at medical institutions should facilitate prompt and appropriate PAC for patients with stroke. Third, the Taiwan National Health Insurance Administration should include PAC in its guidelines for clinical treatment of stroke in order to achieve a broad nationwide improvement in care for these patients. However, further studies are needed to confirm the clinical relevance of the proposed ANN model in terms of its efficacy in predicting prognosis and optimizing medical management for patients with stoke.

For further validation of the significant association observed between PAC and 30-day readmission for stroke, Table 7 compares six relevant studies performed in the United States or Taiwan (39–44). The six studies shared the following features: (1) a relatively large sample size, (2) a mean age of 65 years or more, (3) use of statewide or national datasets, and, most importantly, (4) investigation of 30-day readmission in patients with stroke. As in these previous works, out study demonstrated a significantly lower 30-day stroke readmission rate in a multidisciplinary PAC group compared to a non-PAC group (*P* < 0.001).

**Table 7:** Reported associations between post-acute care (PAC) for stroke and 30-day readmission.

**Authors (country)**	**No. of subjects**	**Mean age**	**Data source**	**Findings**
Present study (Taiwan)	1,476	65.5	Prospective cohort study from six hospitals	Post-acute care (PAC) program was the best predictor of 30-day readmission
Kim et al. (U.S.) (39)	51,863	80.4	Medicare provider analysis and review files	Using Instrumental Variable analysis to control for endogeneity bias, an increase in institutional PAC use was associated with a decrease in 30-day readmission rate by 0.19 percentage points
Kosar et al. (U.S.) (40)	2,044,231	80.2	Medicare provider analysis and review database	In most rural counties, 30-day readmission rates were 0.3 (95% CI, −0.6 to −0.1) percentage points lower in a non-PAC group compared to a PAC group
Raman et al. (U.S.) (41)	1,613	74.4	State inpatient database, California	Clinical predictors of 30-day readmission included comorbidities (e.g., liver disease, hypertension) and discharge to a PAC facility
Li et al. (U.S.) (42)	7,851,430	65~100	Medicare beneficiaries	An increase in quarterly PAC use was significantly (*P* <0.001) associated with a decrease in 30-day risk-standardized readmission rates for acute myocardial infarction, heart failure, and hip/femur fracture
Ramchand et al. (U.S.) (43)	4,850	53.1	National readmissions database	It showed that discharge to inpatient postacute care facility (adjusted odds ratio 1.61, 95% CI 1.07–2.41) was significantly associated with a higher likelihood of 30-day readmission after discharge
Hsieh et al. (Taiwan) (44)	6,839	69.4	National Health Insurance claims datasets	The 30-day readmission rates were 15.5% for the PAC group vs. 30.4% in the non-PAC group

This study has several limitations inherent in a large database analysis. First, the validity of the comparisons in the study is limited by the exclusion of complications associated with stroke rehabilitation outcomes. Second, the analysis was limited to 30-day readmission, which reduces the subset of patients with stroke in which the ANN model is clinically applicable. Third, imbalance between positive and negative outcomes, i.e., class imbalance, is a common problem in analysis of medical data and has not been satisfactorily addressed (45, 46). Further studies are needed to investigate the use of ensemble algorithm for solving the class imbalance problem. Additionally, whether the timing or duration of the stroke treatment is a relevant prognostic predictor of readmission deserves further study. Nevertheless, the results can still be considered valid given the robustness and statistical significance of the results.

## Conclusions

Based on the comparison results in this study, we conclude that the ANN model is superior to the other forecasting models in terms of accuracy in predicting 30-day readmission for stroke after a hospital discharge. The ANN model outperformed the other models in terms of both accuracy and AUROC curve. Using a machine-learning ANN model to obtain an accurate estimate of 30-day readmission for stroke and to identify risk factors may improve the precision and efficacy of management for these patients. Predictors of stroke can be discussed when educating PAC candidates in the expected course of recovery and health outcomes. Although the practical applicability of database studies such as this have been convincingly demonstrated in the literature, future studies can expand the range of clinical variables included in the analysis, which could obtain additional results and potentially improve prediction accuracy. Such data could be vital for developing, promoting, and improving health policies for treating patients with stroke.

## Data Availability Statement

The raw data supporting the conclusions of this article will be made available by the authors, without undue reservation.

## Ethics Statement

The study protocol was approved by the Institutional Review Board at Kaohsiung Medical University Hospital (KMUH-IRB-20140308) and written informed consent was obtained from each participant.

## Author Contributions

Y-CC and H-YS: conceptualization, data curation, formal analysis, investigation, methodology, resources, software, validation, visualization, writing—original draft, and writing—review and editing. J-HC, Y-JY, S-JL, H-FL, C-HL, H-HH, K-WH, and S-CY: data curation, formal analysis, investigation, methodology, resources, software, validation, and visualization. All authors contributed to the article and approved the submitted version.

## Funding

This study was supported by funding from the NSYSU-KMU JOINT RESEARCH PROJECT (NSYSU-KMU 109-P001 and 110-P017), NPUST-KMU JOINT RESEARCH PROJECT (NPUST-KMU 109-P010, 110-P001, and 111-P010), and the Ministry of Science and Technology (MOST 104-2410-H-037-006-SS2, MOST 106-2410-B-037-076, and MOST 108-2410-H-037-006-SS3) in Taiwan. The funders had no role in study design, data collection and analysis, decision to publish, or preparation of the manuscript.

## Conflict of Interest

The authors declare that the research was conducted in the absence of any commercial or financial relationships that could be construed as a potential conflict of interest.

## Publisher's Note

All claims expressed in this article are solely those of the authors and do not necessarily represent those of their affiliated organizations, or those of the publisher, the editors and the reviewers. Any product that may be evaluated in this article, or claim that may be made by its manufacturer, is not guaranteed or endorsed by the publisher.
